# Determination of ileal digestible and apparent metabolizable energy contents of expeller-extracted and solvent-extracted canola meals for broiler chickens by the regression method

**DOI:** 10.1186/s40064-016-2325-z

**Published:** 2016-05-23

**Authors:** Changsu Kong, Olayiwola Adeola

**Affiliations:** Department of Animal Science and Technology, Konkuk University, Seoul, 05029 Republic of Korea; Department of Animal Sciences, Purdue University, West Lafayette, IN 47907-2054 USA

**Keywords:** Broiler chickens, Expeller-extracted canola meal, Ileal digestible energy, Metabolizable energy

## Abstract

The present study was conducted to determine ileal digestible energy (IDE), metabolizable energy (ME), and nitrogen-corrected ME (ME_n_) contents of expeller- (EECM) and solvent-extracted canola meal (SECM) for broiler chickens using the regression method. Dietary treatments consisted of a corn–soybean meal reference diet and four assay diets prepared by supplementing the reference diet with each of canola meals (EECM or SECM) at 100 or 200 g/kg, respectively, to partly replace the energy yielding sources in the reference diet. Birds received a standard starter diet from day 0 to 14 and the assay diets from day 14 to 21. On day 14, a total of 240 birds were grouped into eight blocks by body weight and randomly allocated to five dietary treatments in each block with six birds per cage in a randomized complete block design. Excreta samples were collected from day 18 to 20 and ileal digesta were collected on day 21. The IDE, ME, and ME_n_ (kcal/kg DM) of EECM or SECM were derived from the regression of EECM- or SECM-associated IDE, ME and ME_n_ intake (Y, kcal) against the intake of EECM or SECM (X, kg DM), respectively. Regression equations of IDE, ME and ME_n_ for the EECM-substituted diet were Y = −21.2 + 3035X (r^2^ = 0.946), Y = −1.0 + 2807X (r^2^ = 0.884) and Y = −2.0 + 2679X (r^2^ = 0.902), respectively. The respective equations for the SECM diet were Y = 20.7 + 2881X (r^2^ = 0.962), Y = 27.2 + 2077X (r^2^ = 0.875) and Y = 24.7 + 2013X (r^2^ = 0.901). The slope for IDE did not differ between the EECM and SECM whereas the slopes for ME and ME_n_ were greater (*P* < 0.05) for the EECM than for the SECM. These results indicate that the EECM might be a superior energy source for broiler chickens compared with the SECM when both canola meals are used to reduce the cost of feeding.

## Background

Canola meal is a by-product of canola seed oil extraction and is a good source of essential amino acids for broiler chickens leading to its frequent use in poultry diet formulation as a protein source (Newkirk 2009). There are two extraction methods for obtaining canola oil from canola seed. Solvent extraction is the most common method and uses solvent to improve oil-extraction efficiency, resulting in a meal with <5 % residual oil. On the other hand, the expeller extraction is suggested to be less efficient for oil extraction because the oil is extracted only mechanically, thus leaving more oil (8–15 %) in the meal compared with solvent extraction method (Spragg and Mailer 2007). Moreover, it does not leave any solvent residues which would remain in the solvent-extracted meal. In addition, processing temperature and moisture contents also differ depending on extraction method. The processing conditions for expeller-extracted canola meal (EECM) are less in moisture (<12 % vs. 15–18 %) and higher in temperature (up to 160 °C vs. 95–115 °C) than for solvent-extracted canola meal (SECM), respectively (Newkirk 2009).

Processing conditions for canola oil extraction as well as residual oil in the meal can affect the nutritive values of canola meal (Woyengo et al. 2010b; Khajali and Slominski 2012). It has been reported that EECM had greater amino acid (AA) digestibility and metabolizable energy (ME) than SECM fed to growing pigs (Woyengo et al. 2010a; Maison and Stein 2014). However, there is a dearth of studies which evaluated the nutritive values of EECM for broilers. Woyengo et al. (2010b) reported that doubly extracted EECM compared with SECM fed to broilers had more standardized ileal digestible AA and N-corrected apparent ME (AME_n_) by using the difference method. Toghyani et al. (2014) reported that ileal digestible energy (IDE), apparent ME (AME), and AME_n_ values vary depending on processing conditions and chemical composition of EECM fed to broilers. In view of the dearth of data, the objective of the present study was to determine IDE, ME and ME_n_ of single-extracted EECM and SECM fed to broiler chickens using the regression method.

## Methods

All protocols for the experiment were reviewed and approved by the Purdue University Animal Care and Use Committee.

### Animals and experimental diets

Day-old male broiler chicks of the Ross 308 (Aviagen, Huntsville, AL, USA) strain were obtained from a local hatchery, tagged with identification numbers, and housed in electrically heated battery cages (model SB 4 T, Alternative Design Manufacturing, Siloam Springs, AR, USA) in an environmentally controlled room. Battery brooder temperatures from day 0 to 7 and day 7–14 were kept at 35 and 32 °C, respectively. All birds received a mash standard broiler starter diet from day 0 to 14 (Table 1). On day 14, the birds were weighed individually and 240 birds were grouped into eight blocks by body weight and randomly allocated to five dietary treatments in each block with six birds per cage in a randomized complete block design using the Experimental Animal Allotment Program of Kim and Lindemann (2007). Birds were provided ad libitum access to water and experimental diets from day 14 to 21 and the battery brooder temperatures were maintained at 27 °C.

**Table 1:** Ingredient composition of starter diet fed from d 0 to 14

Item	
Ingredients (g/kg)	
Corn	542.2
Soybean meal	360.0
Soybean oil	50.0
Monocalcium phosphate	16.5
Limestone	16.5
Salt	4.0
Vitamin-mineral premix^a^	3.0
dl-Methionine	3.8
l-Threonine	1.1
l-Lysine HCl	2.9
Total	1000
Calculated nutrients and energy	
Crude protein (g/kg)	226.4
Metabolizable energy (kcal/kg)	3143
Calcium (g/kg)	9.5
Phosphorus (g/kg)	7.2
Non-phytate phosphorus (g/kg)	4.7
Total indispensable amino acids (g/kg)	
Arginine	14.6
Histidine	5.9
Isoleucine	9.2
Leucine	18.9
Lysine	14.3
Methionine	8.3
Methionine + Cysteine	10.8
Phenylalanine	10.5
Phenylalanine + Tyrosine	19.1
Threonine	8.3
Tryptophan	3.0
Valine	10.2

^a^Supplied the following per kilogram of diet: vitamin A, 5484 IU; vitamin D3, 2643 ICU; vitamin E, 11 IU; menadione sodium bisulfite, 4.38 mg; riboflavin, 5.49 mg; d-pantothenic acid, 11 mg; niacin, 44.1 mg; choline chloride, 771 mg; vitamin B12, 13.2 µg; biotin, 55.2 µg; thiamine mononitrate, 2.2 mg; folic acid, 990 µg; pyridoxine hydrochloride, 3.3 mg; I, 1.11 mg; Mn, 66.06 mg; Cu, 4.44 mg; Fe, 44.1 mg; Zn, 44.1 mg; Se, 300 µg

The analyzed chemical composition of EECM and SECM used in the present study are presented in Table 2. Dietary treatments consisted of a corn-SBM reference diet and four assay diets. In the reference diet (Table 3), corn, SBM, corn starch, and soybean oil were used as energy yielding sources. The four assay diets were prepared by supplementing the reference diet with each of canola meals (EECM or SECM) at 100 or 200 g/kg, respectively, to partly replace the energy yielding sources in the reference diet. The ratio of the energy yielding sources remained constant in all treatments to enable determination of the energy value of expeller extracted and solvent extracted canola meals by the regression method (Kong and Adeola 2014).

**Table 2:** Analyzed composition (on a DM basis) of the expeller-extracted canola meal (EECM) and solvent-extracted canola meal (SECM) used in the study

Item	EECM	SECM
DM (g/kg)	846.3	911.8
Gross energy (kcal/kg)	5798	4895
Crude protein (N × 6.25) (g/kg)	398.7	402.0
Ether extract (g/kg)	138.7	24.3
Crude fiber (g/kg)	80.2	83.0
Neutral detergent fiber (g/kg)	233.8	265.3
Acid detergent fiber (g/kg)	181.4	189.5
Calcium (g/kg)	6.5	7.9
Phosphorus (g/kg)	12.4	11.7
Indispensable amino acid (g/kg)		
Arginine	25.6	25.1
Histidine	11.1	10.9
Isoleucine	17.0	16.3
Leucine	29.8	28.8
Lysine	24.0	23.8
Methionine	8.0	7.8
Phenylalanine	16.5	16.1
Threonine	16.9	16.3
Tryptophan	5.6	5.2
Valine	22.3	21.5
Dispensable amino acid (g/kg)		
Alanine	19.1	18.3
Aspartic acid	28.2	27.3
Cysteine	10.4	10.2
Glutamic acid	70.3	69.2
Glycine	21.4	20.3
Methionine	8.0	7.8
Proline	25.6	26.8
Serine	14.7	14.4
Taurine	0.6	0.7
Tyrosine	11.5	11.1

Values presented are from 1 replicate analysis for amino acids and means of duplicate analyses for the other nutrients

**Table 3:** Ingredient composition of experimental diets

Item	Reference diet	EECM (g/kg)		SECM (g/kg)	
		100	200	100	200
Ingredients (g/kg)					
Corn	503.4	450.5	397.7	450.5	397.7
Soybean meal	382	341.9	301.8	341.9	301.8
Soybean oil	40	35.8	31.6	35.8	31.6
Monocalcium phosphate	17.5	17.5	17.5	17.5	17.5
Limestone	14	14	14	14	14
Salt	4	4	4	4	4
Chromic oxide premix^a^	25	25	25	25	25
Vitamin-mineral premix^b^	3	3	3	3	3
dl-Methionine	3	3	3	3	3
l-Lysine HCl	1.1	1.1	1.1	1.1	1.1
Cornstarch	7	4.2	1.3	4.2	1.3
EECM	0	100	200	0	0
SECM	0	0	0	100	200
Total	1000	1000	1000	1000	1000
Analyzed nutrients and energy					
Gross energy (kcal/kg)	4070	4183	4254	4147	4161
Crude protein (N × 6.25) (g/kg)	221.1	247.3	256.9	234.2	252.1

*EECM* expeller-extracted canola meal, *SECM* solvent-extracted canola meal

^a^Prepared as 1 g of chromic oxide mixed with 4 g of cornstarch

^b^Supplied the following per kilogram of diet: vitamin A, 5484 IU; vitamin D3, 2643 ICU; vitamin E, 11 IU; menadione sodium bisulfite, 4.38 mg; riboflavin, 5.49 mg; d-pantothenic acid, 11 mg; niacin, 44.1 mg; choline chloride, 771 mg; vitamin B12, 13.2 µg; biotin, 55.2 µg; thiamine mononitrate, 2.2 mg; folic acid, 990 µg; pyridoxine hydrochloride, 3.3 mg; I, 1.11 mg; Mn, 66.06 mg; Cu, 4.44 mg; Fe, 44.1 mg; Zn, 44.1 mg; Se, 300 µg

### Sample collection and chemical analyses

Excreta samples were collected twice daily from day 18 to 20. During collection, waxed paper was placed in trays under the cages, and excreta on the paper were collected. The excreta samples were pooled per cage over the 2 days and stored in a freezer at −20 °C. On day 21, all birds were euthanized by asphyxiation with carbon dioxide and ileal digesta were collected from the distal two-thirds of ileum by gently rinsing with distilled water. The collected ileal digesta from six birds within a cage were pooled and stored in the freezer at −20 °C.

At the completion of the experiment, ileal digesta and excreta samples were thawed and placed in a forced-air oven at 55 °C for 96 h and ground using a mill grinder (Retsch ZM 100, Retsch GmbH & Co., Haan, Germany). Gross energy (GE) of diets, excreta and ileal digesta samples was determined in adiabatic bomb calorimeter (Parr 1261, Parr Instruments Co., Moline, IL, USA) using benzoic acid as a calibration standard. Dry matter analysis of samples was conducted by drying the samples in a drying oven at 105 °C for 24 h (method 934.01; AOAC 2005). Nitrogen contents of the diets, excreta, and ileal digesta were determined using the combustion method (Model FP2000, Leco Corp., St. Joseph, MI) with EDTA as a calibration standard. Chromium concentrations in the diets, ileal, and excreta samples were determined using the method of Fenton and Fenton (1979).

### Calculations

The coefficients of ileal digestibility and metabolizability of DM, N and energy in the experimental diets were calculated using the index method with chromic oxide as an indigestible index (Kong and Adeola 2014). The IDE and AME contents of experimental diets were then calculated as the product of respective coefficients and the gross energy (kcal/kg). The AME was corrected to zero N retention using the factor of 8.22 kcal/g of N (Hill and Anderson 1958). The IDE, ME or ME_n_ of the test ingredients (SECM and EECM) were calculated using the regression method described in the study by Adeola and Ileleji (2009). Gross energy of the assay diets was corrected for non-energy yielding fractions and then the substitution rates of the canola meals were corrected for energy contributions of energy-yielding ingredients and canola meals in the assay diet. The energy contribution-corrected substitution rate was multiplied with dietary IDE, ME or ME_n_ intake to determine EECM- or SECM-associated corresponding energy intake (kcal). This was then regressed against the intake of EECM or SECM (kg DM) to determine the IDE, ME or MEn (kcal/kg DM) of the canola meal samples using slopes of the regression lines.

### Statistical analysis

Data for the ileal digestibility and total tract retention of energy were analyzed using the GLM procedures of SAS (SAS Institute Inc., Cary, NC, USA). The model included diet and block as the independent variables and individual cage served as the experimental unit. The orthogonal polynomial contrast was used to examine the relationship between energy utilization response criteria and graded concentrations of either EECM or SECM. The EECM- or SECM-associated IDE, ME or MEn intake (kcal) was regressed against the intake of EECM or SECM for cage of six birds, respectively. Slopes and intercepts derived from 3 cages of 0, 100, or 200 g of canola meal substitution in each block were generated using the SLOPE and INTERCEPT functions in Microsoft Office Excel 2010 (Microsoft Corp., Redmond, WA, USA), respectively. The intercept and slope data were analyzed as a one-way ANOVA in a completely randomized design using canola meal type as the independent variable and the intercept or slope as the dependent variable with 1 df for canola meal type and 14 df for the error term (Adeola and Ileleji 2009). In this analysis, experimental unit was a block of 3 cages of 0, 100, or 200 g of canola meal type substitution. Statistical significance level was set at 5 %.

## Results

The CP contents in EECM and SECM were analyzed to be similar (398.7 vs. 402.0 g/kg, on DM basis), which was also reflected in the analyzed values of AA for the respective ingredients. The EECM had greater GE (5789 vs. 4895 kcal/kg, on DM basis) and ether extract (EE) (138.7 vs. 24.3 g/kg, on DM basis) contents than SECM whereas the value of neutral detergent fiber (NDF) was greater in the SECM than in EECM (265.3 vs. 233.8 g/kg). Regardless of the canola type, the body weight gain, feed intake, and feed efficiency of the birds were not influence (P > 0.05) by the supplementation of canola meal to the reference diet (Table 4).

**Table 4:** Growth performance of broilers fed the experimental diets containing expeller-extracted canola meal (EECM) or solvent extracted canola meal (SECM) levels at 0, 100, 200 g/kg

Item	0	EECM		SECM		SEM	*P* value			
		100	200	100	200		L^a^	Q^a^	L^b^	Q^b^
Initial body weight (g)	400	400	400	401	400	0.5	0.858	0.608	0.744	0.273
Final body weight (g)	762	768	724	766	737	14.2	0.077	0.179	0.223	0.367
Body weight gain (g/7 day/bird)	362	368	324	365	337	14.0	0.074	0.168	0.221	0.380
Feed intake (g/7 day/bird)	558	526	514	531	525	19.1	0.124	0.694	0.248	0.661
Feed efficiency (g/kg)	655	699	635	689	642	25.2	0.589	0.099	0.707	0.196

Values are means of five replicate cages with six birds per cage

*SEM* standard error of the mean

^a^Linear (L) and quadratic (Q) contrasts for the EECM

^b^Linear (L) and quadratic (Q) contrasts for the SECM

The data presented in Table 5 show the ileal digestibility and total tract retention of DM, N and energy of experimental diets used in the current study. There were linear (*P* < 0.05) and quadratic (*P* < 0.05) decreases in ileal digestibility of DM and energy as well as IDE as the EECM level in the diets increased from 0 to 200 g/kg. As the SECM substitution into the reference diet increased from 0 to 200 g/kg, all the response criteria measured linearly increased (*P* < 0.05) with the exception of the N retention which was not affected by the substitution of SECM.

**Table 5:** Dry matter (DM), energy, and nitrogen (N) digestibility and total tract retention of broilers fed the experimental diets containing expeller-extracted canola meal (EECM) or solvent extracted canola meal (SECM) levels at 0, 100, 200 g/kg

Item	0	EECM		SECM		SEM	P value			
		100	200	100	200		L^a^	Q^a^	L^b^	Q^b^
Ileal DM digestibility (%)	72.1	66.9	67.1	71.3	67.9	0.598	<0.001	0.002	<0.001	0.086
Total tract DM retention (% of DM intake)	66.9	64.9	62.2	66.3	60.4	1.623	0.057	0.852	0.012	0.200
Ileal N digestibility (%)	82.9	80.8	82.3	82.3	80.5	0.701	0.530	0.046	0.028	0.499
Total tract N retention (% of N intake)	55.8	57.0	54.0	56.6	50.3	2.828	0.655	0.550	0.191	0.325
Total tract N retention (mg/g of DM intake)	22.2	22.7	21.5	22.5	20.0	1.126	0.656	0.550	0.191	0.325
Ileal energy digestibility (%)	76.0	72.1	72.1	75.7	73.1	0.520	<0.001	0.007	0.001	0.087
Total tract energy retention (% energy intake)	73.7	72.1	70.1	73.2	68.3	1.227	0.052	0.933	0.007	0.164
IDE (kcal/kg of DM)	3485	3358	3399	3498	3399	24	0.024	0.013	0.024	0.078
AME (kcal/kg of DM)	3379	3355	3304	3382	3178	57	0.362	0.846	0.024	0.156
AME_n_ (kcal/kg of DM)	3196	3168	3127	3198	3013	49	0.332	0.914	0.018	0.141

*SEM* standard error of the mean, *IDE* ileal digestible energy, *AME* apparent metabolizable energy, *AME*_*n*_ nitrogen-corrected AME

^a^Linear (L) and quadratic (Q) contrasts for the EECM

^b^Linear (L) and quadratic (Q) contrasts for the SECM

**Fig. 1:**
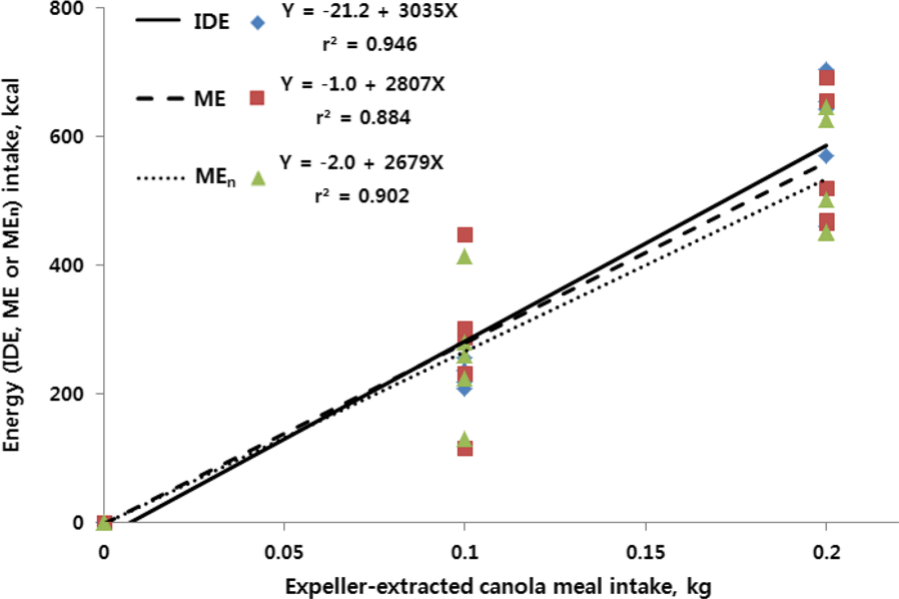
Regression of energy (IDE, ME or ME_n_) intake (Y, kcal) associated with expeller-extracted canola meal (EECM) intake against the intake of EECM (X, kg DM) for birds fed experimental diets from day 14 to 21. Regression equations of IDE, ME and ME_n_ for the EECM-substituted diet were Y = −21.2 + 3035X (r^2^ = 0.946), Y = −1.0 + 2807X (r^2^ = 0.884) and Y = −2.0 + 2679X (r^2^ = 0.902), respectively

**Fig. 2:**
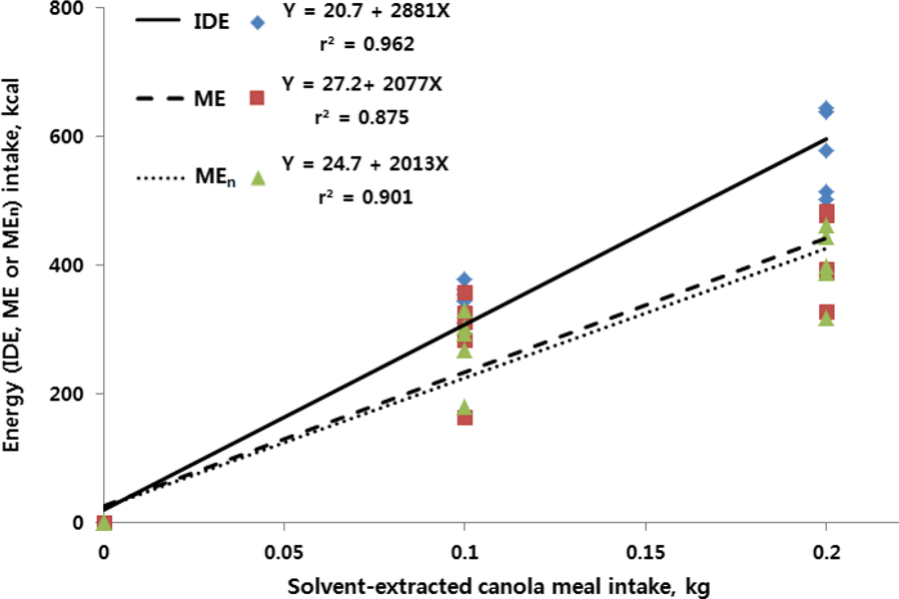
Regression of energy (IDE, ME or ME_n_) intake (Y, kcal) associated with solvent-extracted canola meal (SECM) intake against the intake of SECM (X, kg DM) for birds fed experimental diets from day 14 to 21. Regression equations of IDE, ME and ME_n_ for the SECM-substituted diet were Y = 20.7 + 2881X (r^2^ = 0.962), Y = 27.2 + 2077X (r^2^ = 0.875) and Y = 24.7 + 2013X (r^2^ = 0.901), respectively

The regression of IDE, ME and ME_n_ intake associated with either EECM or SECM against the intake of substituted canola meals depicted in Figs. 1 or 2, respectively. Regression equations of IDE, ME and ME_n_ for the EECM substituted diet were Y = −21.2 + 3035X (r^2^ = 0.946), Y = −1.0 + 2807X (r^2^ = 0.884) and Y = −2.0 + 2679X (r^2^ = 0.902), respectively. The respective equations for the SECM diet were Y = 20.7 + 2881X (r^2^ = 0.962), Y = 27.2 + 2077X (r^2^ = 0.875) and Y = 24.7 + 2013X (r^2^ = 0.901).

Comparisons of slopes and intercepts for the IDE, ME, ME_n_ regressions between the SECM and EECM are presented in Table 6. The slopes for ME and ME_n_ were greater (*P* < 0.05) for the EECM than for the SECM, whereas slope for IDE did not differ between the EECM and SECM.

**Table 6:** Comparison of two types of canola meal for intercepts and slopes of the regressions in the determination of ileal digestible energy (IDE), metabolizable energy (ME) and nitrogen-corrected ME (ME_n_) of expeller- or solvent extracted canola meal

Item	Intercept (kcal)	Slope (kcal/kg)
IDE		
Expeller extracted	−21.2	3035
Solvent extracted	20.8	2881
SEM	5.6	183
*P* value	<0.001	0.568
ME		
Expeller extracted	−1	2807
Solvent extracted	27.4	2077
SEM	19.7	198
*P* value	0.338	0.031
ME_n_		
Expeller extracted	−2.2	2679
Solvent extracted	24.8	2013
SEM	16.4	174
*P* value	0.279	0.027

*SEM* standard error of the mean

## Discussion

The general steps of solvent-extraction method include flaking, cooking, pressing, and solvent-extraction of seeds followed by desolventizing, toasting, and drying of the extracted meal whereas the steps for expeller-extraction method only include flaking, cooking, and pressing of seeds (Spragg and Mailer 2007). The difference in extraction method could influence the composition of two types of canola meal. In the current study, the EECM had 18.4 and 471 % greater GE and EE, respectively, than SECM, which could be due to the difference in extraction method because an additional oil-extraction process by solvent is performed for the SECM. Woyengo et al. (2010b) reported greater GE (5199 vs. 4812 kcal/kg on DM basis) and EE (12.03 vs. 5.54 %, on DM basis) contents in EECM compared with SECM. Moreover the GE and EE contents in EECM used in the previous study was slightly less compared with the present study, which could be attributed to the number of extractions performed (single extraction for the present study vs. double extraction for the previous study) to maximize oil recovery.

The greater NDF content in the SECM compared with the EECM observed in the current study is in agreement with the study by Woyengo et al. (2010b). One of common Maillard reaction products is neutral detergent insoluble nitrogen (Khajali and Slominski 2012) which falls in NDF fraction (van Soest 1994). Maillard reaction can occur during both oil extraction processes due to high temperature of the expeller pressing process for the EECM and desolventizing and toasting processes for the SECM, but the extent of the Maillard reaction may be less for the EECM than for the SECM because the expeller pressing process is faster compared with desolventizing and toasting processes (Landero et al. 2012). The 903 kcal/kg DM greater GE in EECM than in SECM (5798 vs. 4895) is fully accounted for by the 114 g/kg DM greater EE in EECM than in SECM (138.7 vs. 24.3) and the 31.5 g/kg DM lower NDF in EECM than in SECM (238.8 vs. 265.3) using gross energies of 9.2 and 4.5 kcal/g for EE and NDF.

Regardless of the type of canola meals, the influences by the addition of graded canola meal were not observed. These results might be attributed to the short experimental period in which the effect of treatments on the growth performance may not be obvious (Woyengo et al. 2010b).

Substitution of two canola meals at 100 or 200 g/kg of diet for the energy supplying ingredients (corn, soybean meal, soybean oil, and cornstarch) in the reference diet linearly decreased ileal DM and energy digestibility as well as the IDE of diet. This may be attributed to the level of fiber in both canola meals used in the current study because both EECM and SECM had relatively greater contents of fiber compared with corn and SBM (NRC 2012). Due to the physical presence of fiber in the gastrointestinal tract, fiber may have a detrimental influence on the utilization of nutrients in broiler chickens (Pettersson and Aman 1989; Choct and Annison 1990). The physical barrier of the cell walls of fiber can encapsulate potentially available nutrients. Furthermore, the viscous properties of fiber may interfere with the digestion process and thereby reduce the digestibility of other nutrients (Choct and Annison 1992; Steenfeldt 2001). Consequently, the fiber components have negative impact on the digestibility of nutrients in the broiler chickens and lead to lower energy utilization. High dietary fiber may increase the passage rate of digesta (Khajali and Slominski 2012), which adversely affect the digestibility of nutrient.

In the current study, the EECM compared with SECM had 35 or 33 % more ME (2807 vs. 2077 kcal/kg) or ME_n_ (2679 vs. 2013 kcal/kg) estimated by the regression method, respectively. Using the difference method, Woyengo et al. (2010b) also reported greater AME (3039 vs. 2005 kcal/kg, on DM basis) and AME_n_ (2694 vs. 1801 kcal/kg, on DM basis) contents in EECM than in SECM fed to broilers. This in part could result from the difference in the fat content between the EECM and SECM. The concentration of ether extract in EECM used in the present study or the study by Woyengo et al. (2010b) was 471 (13.87 vs. 2.43 %) or 217 % (5.54 vs. 12.03 %) greater than in SECM, respectively.

The significant differences in ME or ME_n_ between the SECM and EECM were observed in the present study whereas the difference in the IDE was not significant. The reason for these results was not clear. Speculatively, difference in the fermentation of undigested dietary fibrous contents might have contributed to such difference in ME values. Because microbes in the ceca of broiler chickens are able to ferment undigested dietary fiber contents entering from the ileum into short chain fatty acids which could be absorbed by broiler chickens and used as energy (Sugahara et al. 2004).

## Conclusion

In conclusion, the results showed that the EECM used in the current study had more fat as well as less NDF and consequently more GE content compared with the SECM. In addition, the ME and ME_n_ contents of the EECM were greater than those of the SECM, which indicates that the EECM might be a superior energy source for broiler chickens compared with the SECM when both canola meals are used to reduce the cost of feeding.
